# Cost-Effectiveness of Noninvasive Colorectal Cancer Screening in Community Clinics

**DOI:** 10.1001/jamanetworkopen.2024.54938

**Published:** 2025-01-16

**Authors:** Pedro Nascimento de Lima, Laura Matrajt, Gloria Coronado, Anne L. Escaron, Carolyn M. Rutter

**Affiliations:** 1 Engineering and Applied Sciences Department, RAND, Arlington, Virginia; 2 Fred Hutchinson Cancer Research Center, Vaccine and Infectious Diseases Division, Seattle, Washington; 3 Applied Mathematics Department, University of Washington, Seattle; 4 University of Arizona Cancer Center, Tucson; 5 Institute for Health Equity, AltaMed Health Services Corporation, Los Angeles, California; 6 Fred Hutchinson Cancer Research Center, Hutchinson Institute for Cancer Outcomes Research and Biostatistics Program, Public Health Sciences Division, Seattle, Washington

## Abstract

**Importance:**

Several noninvasive tests for colorectal cancer screening are available, but their effectiveness in settings with low adherence to screening and follow-up colonoscopy is not well documented.

**Objective:**

To assess the cost-effectiveness of and outcomes associated with noninvasive colorectal cancer screening strategies, including new blood-based tests, in a population with low adherence to screening and ongoing surveillance colonoscopy.

**Design, Setting, and Participants:**

The validated microsimulation model used for the decision analytical modeling study projected screening outcomes from 2025 to 2124 for a simulated cohort of 10 million individuals aged 50 years in 2025 and representative of a predominantly Hispanic or Latino patient population served by a Federally Qualified Health Center in Southern California. The simulated population had low adherence to first-step noninvasive testing (45%), second-step follow-up colonoscopy after an abnormal noninvasive test result (40%), and ongoing surveillance colonoscopy among patients with high-risk findings at follow-up colonoscopy (80%).

**Exposures:**

Colorectal cancer screening strategies included no screening, an annual or biennial fecal immunochemical test, a triennial multitarget stool DNA test, and a triennial blood-based test. Using a blood-based test was assumed to increase first-step adherence by 17.5 percentage points.

**Main Outcomes and Measures:**

Outcomes included colorectal cancer incidence and mortality, life-years gained and quality-adjusted life-years gained relative to no screening, costs, and net monetary benefit assuming a willingness to pay of $100 000 per quality-adjusted life-year gained.

**Results:**

Under realistic adherence assumptions, a program of annual fecal immunochemical testing was the most effective and cost-effective strategy, yielding 121 life-years gained per 1000 screened individuals and a net monetary benefit of $5883 per person. Triennial blood testing was the least effective, yielding 23 life-years gained per 1000, and was not cost-effective, with a negative net monetary benefit. Annual fecal immunochemical testing with 45% first-step adherence and 80% adherence to follow-up and surveillance colonoscopy yielded greater benefit than triennial blood testing with perfect adherence (88 vs 77 life-years gained per 1000).

**Conclusions and Relevance:**

This study suggests that in a federally qualified health care setting, prioritizing the convenience of blood tests over less costly and more effective existing stool-based tests could result in higher costs and worse population-level outcomes. Novel screening modalities should be carefully evaluated for performance in community settings before widespread adoption.

## Key Points

**Question:**

How effective and cost-effective are stool- and blood-based colorectal cancer screening strategies when screening adherence is low?

**Findings:**

This decision analytical model using a validated microsimulation model to project screening outcomes for a simulated cohort of 10 million 50-year-old individuals representative of a predominantly Hispanic or Latino patient population found that annual screening with fecal immunochemical tests (FITs) was the most effective and least costly strategy. Triennial blood-based screening was the least effective and most costly strategy.

**Meaning:**

This study suggests that when screening adherence is low, FIT is associated with the most benefit at the lowest cost and that increasing receipt of colonoscopy after an abnormal FIT result could further improve patient outcomes.

## Teaser

This decision analytical model assesses the effectiveness and cost-effectiveness of noninvasive colorectal cancer (CRC) screening strategies in a population with low adherence to screening and ongoing surveillance colonoscopy.

## Introduction

Federally qualified health centers (FQHCs) serve populations facing longstanding multilevel barriers to colorectal cancer (CRC) screening.^1^ With constrained resources, FQHCs often rely on noninvasive CRC screening tests recommended by the US Preventive Services Task Force (USPSTF).^2^ A program of annual fecal immunochemical test (FIT) screening is an effective way to improve CRC outcomes and is consistent with approaches adopted by large integrated health care systems.^3,4^ Noninvasive screening is a 2-step process: the first step is completion of the noninvasive test; results determine which patients proceed to the second step, completion of a follow-up colonoscopy. Finally, patients with high-risk noncancerous findings at their follow-up colonoscopy enter a program of adenoma surveillance.^5^ In the US, an annual FIT is the most common noninvasive screening test.^6^

Before the COVID-19 pandemic, adherence to FIT screening in FQHCs was near 45%.^7^ After the pandemic, FIT screening rates dropped as low as 30% in some FQHCs,^8^ and many have not yet returned to prepandemic CRC screening levels.^9^ As of 2020, about 3.6 million people served by FQHCs were not up to date with CRC screening.^10^

To address low rates of FIT screening, many health care systems offered triennial multitarget stool DNA (mt-sDNA) screening.^11^ Blood-based biomarker screening tests are now emerging as another noninvasive screening method that could improve adherence.^12,13^ However, modeling studies project that blood tests would be less effective than existing stool-based tests.^14,15^ This is because screening, even with noninvasive tests, prevents CRC by detecting and removing precursor lesions,^16,17^ reducing incidence, and lowering treatment costs. Blood tests do not detect precursor lesions.

Previous modeling studies found that blood-based test screening would not be effective when population-level screening rates reflect national levels.^14,18^ The effectiveness and cost-effectiveness of noninvasive tests are not well studied in settings where adherence is low for both the noninvasive screening and follow-up colonoscopy. This study used the Colorectal Cancer Simulated Population model for Incidence and Natural history (CRC-SPIN)^19,20^ to assess the effectiveness and cost-effectiveness of noninvasive CRC screening strategies, including screening with FIT, an mt-sDNA test, or a blood-based test in an FQHC setting with low adherence rates.

## Methods

### Setting

This analysis focuses on projecting outcomes for a predominately Hispanic or Latino population served by a large, urban FQHC system that operates 25 medical clinics in Southern California. In 2022, this system served more than 245 000 patients, including over 65 000 patients who were age eligible for CRC screening (50-75 years); 46.8% of these age-eligible patients were up to date with CRC screening.^21^ In 2018, 33.3% of the FQHC’s patients with an abnormal FIT result completed a follow-up colonoscopy within 12 months.^22^ Overall, completion of follow-up colonoscopy within 6 to 12 months of an abnormal FIT result ranges from 18% to 57% across FQHCs.^23,24^ In 2022, the Southern California FQHC began offering triennial mt-sDNA tests with a goal of improving the fraction of patients who are up to date with CRC screening. The Fred Hutchinson Cancer Center institutional review board waived human participant approval because this simulation study did not use patient data. This study followed the Consolidated Health Economic Evaluation Reporting Standards (CHEERS) reporting guideline.

### Microsimulation Model

The CRC-SPIN model^19,20^ is an established microsimulation model that is part of the Cancer Intervention and Surveillance Modeling Network (CISNET) and has been used to support screening recommendations by the USPSTF.^25,26^ CRC-SPIN simulates the natural history of CRC via the adenoma-carcinoma pathway and has been calibrated to match a set of empirical targets derived from the literature and including US CRC incidence data.^20^

### Simulated Cohort

Our analyses focus on a simulated cohort of 10 million individuals born in 1975 who turn 50 years old in 2025. To simulate expected CRC incidence in the lower-risk, predominantly Hispanic population served by the FQHC, we recalibrated CRC-SPIN so that the decision analytic model accurately predicted the 2017-2021 CRC incidence for Hispanic adults aged 45 to 49 years in the Los Angeles Surveillance, Epidemiology, and End Results Registry (eMethods 1 in Supplement 1).^27^ We used the 45- to 49-year age range for calibration because this population was not eligible for screening when the data were collected. The recalibrated model assumes that differences in CRC incidence across ethnicities and cohorts stem from differences in adenoma prevalence rather than differential disease progression.

### Screening Strategies

We simulated 4 screening strategies: no screening, annual FIT, biennial FIT,^4,5^ triennial mt-sDNA test,^28^ and a triennial blood test (Shield; Guardant Health).^29^ Test sensitivity and specificity assumptions (Table 1) are the same as those used in recent analyses that estimated effectiveness under perfect adherence to screening recommendations^14,15^ and are based on published estimates for FIT, mt-sDNA, and Shield tests.^28,29^ We simulated 2 strategies that are not USPSTF recommended: (1) biennial FIT, which is consistent with screening programs in other countries and is recommended by the American College of Physician,^30,31^ and a triennial blood test based on the Centers for Medicare & Medicaid Services 2023 National Coverage Determination.^32^ We simulated all screening strategies beginning at 50 years of age, because uptake of screening among people 45 to 49 years of age remains low.^33^ For all scenarios, we simulated screening up to 75 years of age and continued adenoma surveillance up to 85 years of age.

**Table 1.** Sensitivity, Specificity, and Realistic Adherence Assumptions for Each Screening Strategy

Strategy	Sensitivity, %				Specificity (no cancer or adenomas), %	Adherence, %	
	Colorectal cancer	Adenomas, by size				First step: noninvasive test	Second step: follow-up colonoscopy
		<5 mm	6-9 mm	≥10 mm			
Annual FIT	74	5	15	24	96	45	40
Biennial FIT	74	5	15	24	96	45	40
Annual FIT+	74	5	15	24	96	45	80
Triennial mt-sDNA test	94	11	31	42	91	45	40
Triennial blood-based test	83	10	10	10	90	62.5	40

Abbreviations: FIT, fecal immunochemical test; FIT+, FIT screening that assumes 80% of individuals with an abnormal result complete follow-up colonoscopy; mt-sDNA, multitarget stool DNA.

### Adherence to Screening, Follow-Up Colonoscopy, and Surveillance

Analyses focus on 2 primary screening adherence scenarios: perfect adherence (100%), which was used as a reference, and realistic adherence (45%) to a noninvasive screening test that reflected prepandemic screening levels observed in FQHCs.^34^ We assumed that realistic adherence to CRC blood tests would be 62.5%, 17.5 percentage points higher than adherence to FIT as estimated by a recent study of patient adherence in an integrated care setting.^12^ Adherence was simulated at the per-test level. For example, 45% adherence to screening was simulated by applying a 0.45 probability of completing screening each time a test was offered, based on the screening schedule (annually, biennially, or triennially). We made the simplifying assumption that the probability of completing screening is independent of screening history. This allows patients to intermittently miss tests.

Follow-up colonoscopy is required to complete screening after abnormal findings from a noninvasive screening test (FIT, mt-sDNA test, or blood test). For realistic adherence scenarios, we assumed that individuals with an abnormal noninvasive test result have a 0.40 probability of completing a follow-up colonoscopy, reflecting rates observed in FQHCs.^22,23,24^ To understand how a program focused on increasing receipt of follow-up colonoscopy after an abnormal FIT result could affect screening benefits, we also simulated an annual “FIT+” adherence scenario with an 0.80 probability of completing a follow-up colonoscopy, reflecting follow-up rates observed in integrated health care systems.^3,35^ We made the simplifying assumption that the probability of receiving a follow-up colonoscopy is independent of screening history and screening modality. Individuals who do not complete follow-up colonoscopy are assumed to return to screening.

Individuals with findings at colonoscopy enter adenoma surveillance, following the 2020 US Multi-Society Task Force on Colorectal Cancer recommendations.^5^ Real-world adherence to surveillance is known to be imperfect.^36^ Realistic adherence scenarios assumed that individuals have a 0.80 probability of receiving colonoscopy at each recommended surveillance interval.

### Outcomes

Clinical outcomes were the lifetime number of CRC cases and deaths, the number of triage tests received, and the number of colonoscopies associated with screening (ie, follow-up and surveillance colonoscopies, but not diagnostic colonoscopies). Effectiveness measures were life-years gained (LYG) and quality-adjusted life-years (QALYs) gained relative to no screening, calculated over the cohort’s remaining lifetime (from 2025 forward). Quality-adjusted life-years were adjusted for utility loss from both CRC screening and treatment. Utility weights are provided in eMethods 2 and eTable in Supplement 1, and correspond to previous analyses.^14,15^ Clinical outcomes are presented as rates or counts per 1000 50-year-old individuals eligible for screening in 2025, that is, alive and free from diagnosed CRC at the beginning of 2025.

Colorectal cancer care costs were calculated from the health care perspective and included costs of screening, follow-up and surveillance colonoscopy, and diagnosis and treatment. Cost estimates (eTable in Supplement 1) were drawn from the literature,^14,15,37^ as in previous analyses.^14,15^ For mt-sDNA tests, blood tests, and follow-up colonoscopy, costs were accrued only for completed tests; missed tests had no cost. FIT costs were realized each time the test was offered, regardless of completion, assuming mailed distribution. Costs do not include a health care visit that may precipitate screening. The net cost of each screening program is the difference in the total cost relative to no screening. Costs for annual FIT+ screening, with 80% adherence to follow-up colonoscopy, did not include the cost of a hypothetical intervention to achieve increased adherence to follow-up colonoscopy. Costs are reported as mean costs per person. Cost-effectiveness analyses discount both QALYs gained and costs at 3% per year.

Willingness to pay (WTP) refers to how QALYs are valued and determines the cost-effectiveness and net monetary benefit (NMB) of interventions. Analyses use a $100 000 WTP per QALY threshold, a widely used benchmark in cost-effectiveness literature.^38^ The NMB was calculated for each screening strategy relative to no screening using the formula: NMB = (QALYs gained × WTP) − net costs.^39^ The NMB can be interpreted as the mean value of CRC screening, accounting for all benefits and costs incorporated in the analysis. The difference between the NMB of 2 strategies reveals the loss of benefit caused by choosing a suboptimal strategy. A positive NMB means that a screening regimen is cost-effective relative to no screening at the specified WTP threshold. We report NMB per person. Sensitivity analyses examined the cost-effectiveness using a $150 000 WTP threshold and cost-effectiveness of blood tests assuming a $445 per-test cost, approximately half of current published costs of $895 for the Shield test.^18^

## Results

Projected clinical outcomes are shown in Table 2 and the Figure. Without screening, projected lifetime CRC cases in the target population (alive and without diagnosed CRC at 50 years of age) was 66.0 per 1000 individuals, leading to 27.3 CRC deaths. Every screening scenario reduced CRC cases and deaths and resulted in LYG, and every screening modality was more effective with perfect adherence. For both perfect and realistic screening scenarios, annual FIT was the most effective scenario, followed by triennial mt-sDNA testing, then biennial FIT. Triennial blood testing was the least effective screening scenario, yielding 23 LYG per 1000. Realistic adherence to annual FIT screening with 80% adherence to follow-up colonoscopy (annual FIT+) was more effective than a triennial blood test with perfect adherence, yielding fewer CRC cases (32.0 vs 37.7) and CRC deaths per 1000 (11.3 vs 13.0) and more LYG per 1000 (183 vs 167), while requiring fewer follow-up and surveillance colonoscopies per 1000 (816 vs 1144). Realistic adherence to annual FIT alone yielded 121 LYG per 1000.

**Table 2.** Projected CRC Outcomes for Screening Programs Based on a FIT, mt-sDNA Test, or a Blood-Based Test^a^

Screening	CRC		LY, No.	LYG, No.	Triage tests, No.	Colonoscopies, No.
	Cases, No.	Deaths, No.				
No screening	66.0	27.3	31 535	Reference	0	0
Perfect adherence^b^						
Annual FIT	17.4	5.3	31 796	261	16 075	1520
Triennial mt-sDNA test	19.6	6.4	31 779	245	6254	1434
Biennial FIT	24.6	8.1	31 760	225	9534	1105
Triennial blood-based test	37.7	13.0	31 702	167	6565	1144
Realistic adherence^c^						
Annual FIT+	32.0	11.3	31 718	183	8837	816
Annual FIT	43.4	16.6	31 656	121	9529	500
Triennial mt-sDNA test	49.1	19.6	31 620	85	3432	388
Biennial FIT	52.6	20.9	31 607	72	5026	286
Triennial blood-based test	57.0	22.6	31 589	54	4816	330

Abbreviations: CRC, colorectal cancer; FIT, fecal immunochemical test; FIT+, screening that assumes 80% of individuals with an abnormal result complete follow-up colonoscopy; LY, life-years; LYG, life-years gained; mt-sDNA, multitarget stool DNA.

^a^ All estimates are lifetime numbers per 1000 simulated 50-year-old individuals alive and free from diagnosed CRC in 2025. Life-years and LYG are relative to no screening. Colonoscopies exclude diagnostic examinations.

^b^ Perfect adherence scenarios assumed 100% adherence to screening, follow-up colonoscopy, and surveillance colonoscopy.

^c^ Realistic adherence scenarios assumed 45% adherence to screening for FIT and mt-sDNA test and 62.5% adherence for a blood-based test, 40% adherence to follow-up colonoscopy after an abnormal screening test, with the exception of FIT+, which assumed 80% adherence to follow-up colonoscopy, and 80% adherence to surveillance colonoscopy at recommended intervals.

**Figure.**
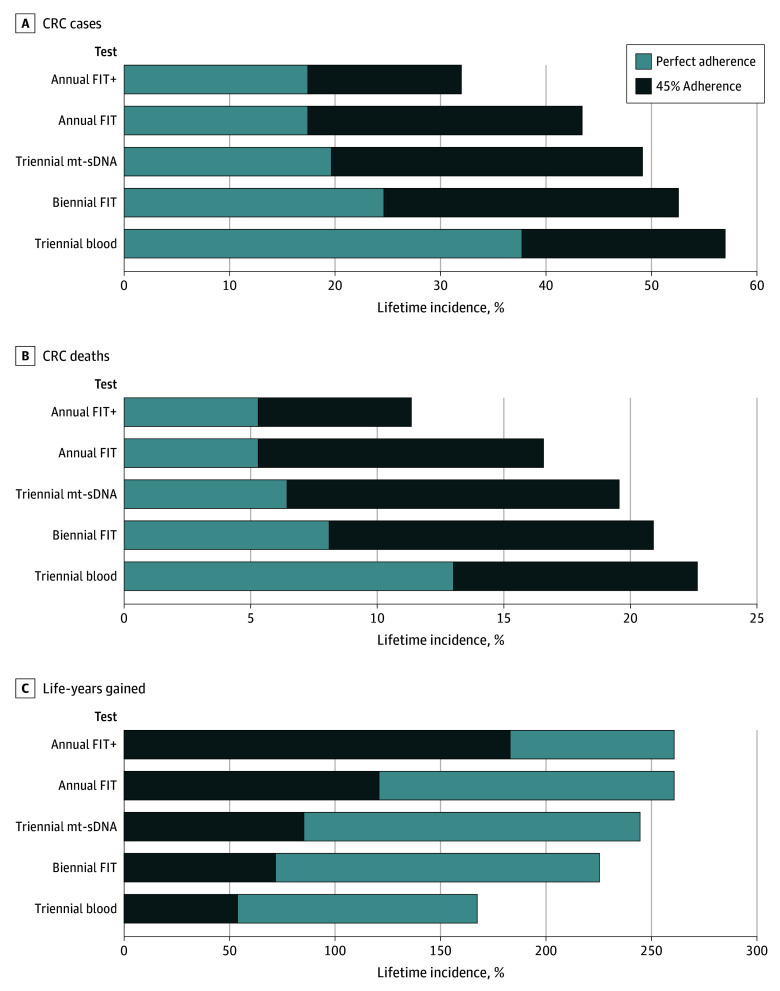
Benefits Associated With Screening Programs for Noninvasive Tests Under Perfect and Realistic Screening Scenarios Projected for a simulated Hispanic cohort that is 50 years of age in 2025. All estimates are lifetime numbers per 1000 simulated 50-year-old individuals alive and free from diagnosed cancer in 2025. Life-years and life-years gained are relative to no screening. Perfect adherence assumed 100% adherence to screening, follow-up colonoscopy, and surveillance colonoscopy. Realistic adherence assumed 45% adherence to screening for fecal immunochemical test (FIT) and multitarget stool DNA (mt-sDNA) and 62.5% adherence for a blood-based test, 40% adherence to follow-up colonoscopy after an abnormal screening test, with the exception of FIT+ (receipt of follow-up colonoscopy after an abnormal FIT result), which assumes 80% adherence to follow-up colonoscopy and 80% adherence to surveillance colonoscopy at recommended intervals.

Projected cost-effectiveness outcomes are shown in Table 3. Without screening, the lifetime mean projected cost of CRC care, which is entirely attributable to diagnosis and treatment, was $4873 per person. Every screening scenario reduced treatment costs and yielded QALY gains, but only FIT-based screening yielded net cost savings. Perfect adherence to screening resulted in lower net costs, more QALYs gained, and greater NMB. Annual FIT resulted in the most QALYs gained, followed by triennial mt-SDNA tests and biennial FIT. Triennial blood tests yielded the least QALYs gained. Annual FIT testing yielded the largest cost savings and highest NMB, indicating the greatest value across tests examined. Biennial FIT testing had lower net costs than triennial mt-sDNA tests and a higher NMB, with larger differences for perfect adherence scenarios. Triennial blood testing had the highest net costs and lowest NMB. Assuming realistic adherence, triennial blood tests were not cost-effective relative to no screening.

**Table 3.** Projected Cost-Effectiveness Outcomes^a^

Screening	Benefits per thousand		Mean costs per person, $					Net monetary benefit per person, $
	QALYs	QALYs gained	Triage test	Colonoscopy	Treatment	Total	Net	
No screening	19 909	Reference	0.00	0.00	4873	4873	Reference	Reference
Perfect adherence^b^								
Annual FIT	20 040	131	300	1588	1781	3669	−1204	14 313
Triennial mt-sDNA test	20 031	122	2588	1492	1999	6079	1206	11 001
Biennial FIT	20 020	111	178	1140	2430	3748	−1125	12 218
Triennial blood-based test	19 986	77	4397	1145	3546	9088	4215	3485
Realistic adherence^c^								
Annual FIT+^d^	19 997	88	360	823	3003	4186	−687	9476
Annual FIT	19 965	56	385	492	3730	4608	−265	5883
Triennial mt-sDNA test	19 949	40	1396	381	3984	5761	888	3118
Biennial FIT	19 942	33	206	282	4221	4708	−165	3473
Triennial blood-based test	19 932	23	3182	324	4523	8029	3156	−825

Abbreviations: FIT, fecal immunochemical test; FIT+, screening that assumes 80% of individuals with an abnormal result complete follow-up colonoscopy; mt-sDNA, multitarget stool DNA; QALYs, quality-adjusted life-years.

^a^ Costs and QALYs are discounted at 3% per year. Quality-adjusted life-years gained, net costs, and net monetary benefit is relative to no screening. Colonoscopy costs count the costs of follow-up and surveillance colonoscopy, while diagnostic colonoscopies are included in treatment costs. Costs are in 2021 dollars. Net monetary benefit assumes a $100 000 willingness-to-pay threshold.

^b^ Perfect adherence scenarios assumed 100% adherence to screening, follow-up colonoscopy, and surveillance colonoscopy.

^c^ Realistic adherence scenarios assumed 45% adherence to screening for FIT and mt-sDNA test and 62.5% adherence for a blood-based test, 40% adherence to follow-up colonoscopy after an abnormal screening test, with the exception of FIT+, which assumed 80% adherence to follow-up colonoscopy, and 80% adherence to surveillance colonoscopy at recommended intervals.

^d^ Costs for annual FIT+ screening, which increases the adherence to the second step, follow-up colonoscopy, do not include the cost of an intervention to increase receipt of follow-up colonoscopy.

The NMB of annual FIT with realistic adherence was greater than the NMB of a triennial blood test with perfect adherence ($5883 vs $3485 per person) (Table 3). Moreover, the NMB of annual FIT with realistic (45%) screening adherence and 80% follow-up colonoscopy adherence (annual FIT+) was $9476. Compared with a blood test with perfect adherence, annual FIT+ would result in 11 QALYs gained per 1000 and $4902 lifetime net cost savings per person. (QALYs gained and net costs are based on subtracting QALYs and net costs for a perfect blood test scenario (77 QALYs and $4215) from those for an annual FIT+ scenario (88 QALYs and –$687.) This means that, even if we were able to achieve perfect adherence to the blood test, it would be rational to instead invest a mean of $5991 per person (over their lifetime) to increase adherence to follow-up colonoscopy from 40% to 80%. Incremental cost-effectiveness ratios were not calculated because annual FIT was both the least expensive and most effective strategy and annual FIT was cost-saving.

With a $150 000 WTP threshold, triennial blood testing with imperfect adherence was cost-effective, with an NMB of $340 per person. Assuming a $150 000 WTP threshold also reduced differences in the NMB of biennial FIT ($17 764) and triennial mt-sDNA test ($17 104) with perfect adherence and resulted in equivalent NMB from biennial FIT and triennial mt-sDNA test with realistic adherence (approximately $5125 for each).

Reducing the cost of a blood test would lower the per-person lifetime cost of the testing (to $2199 under perfect adherence and $1591 under realistic adherence); the NMB of a blood test, assuming a $100 000 WTP threshold, would increase to $5684 under perfect adherence and $766 under realistic adherence, making the blood test cost-effective relative to no screening. Even at this lower cost, it would be rational to adopt annual FIT screening and invest a mean of $3792 per person (over their lifetime) on an intervention that could increase adherence to follow-up colonoscopy from 40% to 80% because it would result in more QALYs gained.

## Discussion

This simulation study examines the benefits, costs, and cost-effectiveness of noninvasive screening tests when adherence to this multistep screening process reflects adherence rates in populations receiving care at FQHCs. Stool-based tests were a cost-effective screening approach, even with low adherence to both noninvasive screening and follow-up colonoscopy. Triennial mt-sDNA testing and biennial FIT yielded lower benefits at a higher cost but were cost-effective relative to no screening. Blood tests yielded the least benefit at the highest costs, due to the combination of their high per-test cost and low sensitivity to detect advanced adenomas. In a population with low adherence to CRC screening, triennial blood-based tests were only cost-effective relative to no screening when at a higher WTP threshold of $150 000 or if the per-test cost were halved, from $895 to $445.

Previous studies found that CRC blood tests could only be cost-effective relative to annual FIT or decennial colonoscopy if they had higher (>40%) sensitivity to detect advanced adenomas and a lower per-test cost (<$125).^15^ Enhancing the sensitivity to detect advanced adenomas using a blood test is a longstanding challenge.^40^ New blood tests can accurately detect cancer,^29^ but not advanced adenomas.^41^ Some have argued that higher adherence to blood tests could compensate for lower detection of advanced adenomas.^42^ Our findings counter this argument. Under realistic screening assumptions, a blood-based test was not cost-effective relative to stool-based tests, even with a realistic adherence advantage.^12^ Furthermore, a FIT program with low (45%) FIT adherence and high (80%) adherence to follow-up colonoscopy would have greater benefit at a lower cost than a blood-based screening program with perfect (100%) adherence to screening, follow-up colonoscopy, and ongoing surveillance colonoscopy. This underscores the value of improving adherence to follow-up colonoscopy to realize the benefits and cost-effectiveness of FIT screening.

### Limitations

There are several limitations to this study. First, modeling screening behaviors necessarily simplifies a complex and poorly observed process. Adherence rates were based on observed FQHC CRC screening rates, and we simulated adherence at the test level, allowing individuals to miss multiple tests. However, we simplified the process by assuming a single level of screening adherence over time, based on a prespecified screening schedule, using a single modality. Second, because CRC screening results in short-term costs and long-term benefit, we accrued costs and benefits for the lifetime of patients, although many individuals will move from the FQHC system on reaching Medicare eligibility at 65 years of age and may increase screening adherence with more consistent insurance benefits. Another limitation is that our model did not incorporate the serrated CRC pathway,^43,44^ which could reduce the preventive benefit estimated for stool-based tests, especially FIT. Projected costs and benefits associated with blood tests would not change, because blood tests for CRC do not detect precursor lesions in the serrated pathway. Although considerable uncertainty remains about the serrated pathway, a recent comparative effectiveness analysis of CRC screening using a model that included the serrated pathway found that inclusion of the serrated pathway had little effect on the relative benefit of screening tests.^45^

Blood-based screening could be cost-effective relative to no screening,^14^ suggesting that it could be offered just to patients who refuse FIT or mt-sDNA test. However, it would likely be difficult to limit use to these individuals who are reluctant to undergo FIT or mt-sDNA testing. In addition, while blood tests may increase adherence to noninvasive CRC screening, there is no reason to believe first-step screening with a blood test would affect adherence to the second critical screening step, receipt of follow-up colonoscopy. This is especially true in populations that face multiple barriers to follow-up colonoscopy.^1^ Given the expense of blood-based tests, it may be more effective to spend limited health care dollars on patient education materials and navigation to support receipt of follow-up colonoscopy.^46^

## Conclusions

This decision analytical model found that annual FIT was projected to be the most effective and cost-effective noninvasive CRC screening modality, even with low adherence rates observed among patients receiving care at FHQCs. Setting health policy based on increasing adherence to noninvasive screening, without considering effectiveness and adherence to follow-up colonoscopy, could waste health care resources and result in inferior patient outcomes. The additional resources needed to pay for blood tests could otherwise be used to improve population-level health, including navigation for patients with abnormal FIT results to improve receipt of follow-up colonoscopy.^47^ In addition, a program of triennial blood tests would result in higher CRC incidence and more CRC deaths than a program of annual FIT screening. However, if health care systems do adopt blood tests, they may be rewarded for better population-level screening adherence, improving their performance metrics.^48^ Steering populations with low screening adherence toward less effective but more convenient tests could widen disparities.

## Notes

Supplement 1. **eMethods 1.** Colorectal Cancer Risk Calibration **eMethods 2.** Costs and Disutility Weights **eTable.** Cost and Disutility From Screening Tests, CRC Care, and Colonoscopy Complications
eReferences.

Supplement 2.
Data Sharing Statement
