# Supplementary material for: Cost-Effectiveness of Noninvasive Colorectal Cancer Screening in Community Clinics
Source: JAMA Netw Open. 2025 Jan 16;8(1):e2454938. doi: 10.1001/jamanetworkopen.2024.54938 (PMC11739995; doi:10.1001/jamanetworkopen.2024.54938)
Supplement: Supplement 1. — eMethods 1. Colorectal Cancer Risk Calibration eMethods 2. Costs and Disutility Weights eTable. Cost and Disutility From Screening Tests, CRC Care, and Colonoscopy Complications eReferences. [file jamanetwopen-e2454938-s001.pdf]

Supplementary Online Content

Nascimento de Lima P, Matrajt L, Coronado G, Escaron AL, Rutter CM, et al. Cost-effectiveness of noninvasive colorectal cancer screening in community clinics. *JAMA Netw Open.* 2025;8(1):e2454938.
doi:10.1001/jamanetworkopen.2024.54938

eMethods 1. Colorectal Cancer Risk Calibration

eMethods 2. Costs and Disutility Weights

eTable. Cost and Disutility From Screening Tests, CRC Care, and Colonoscopy Complications

eReferences.

This supplementary material has been provided by the authors to give readers additional information about their work.

© 2025 Nascimento de Lima P et al. *JAMA Network Open.*

eMethods 1. Colorectal Cancer Risk Calibration

Colorectal cancer (CRC) risk in the Hispanic population covered by the Los Angeles Surveillance, Epidemiology, and End Results (SEER) registry was lower than in the Non-Hispanic White population in the same region. For example, in 2017-2021 CRC Incidence in the SEER 22 dataset was 27.5 cases per 100,000 45-49 year olds for the Hispanic population, and it was 33.5 cases per 100,000 45-49 year olds for the non-Hispanic White population.[1] The target population served by the federally qualified health center (FQHC) of focus in this study is predominantly Hispanic. Hence, an approach is needed to ensure that the model appropriately reflects CRC risks and screening benefits to be observed in this population.

Consistent with the approach used in prior studies,[2,3] we calibrated the overall adenoma incidence risk in the CRC-SPIN model[4] such that the CRC risk projected by the model in an unscreened population aged 45-49 years matches the absolute CRC risk observed in the SEER Los Angeles registry for the Hispanic population (27.5 cases per 100,000 45-49 year olds).

© 2025 Nascimento de Lima P et al. *JAMA Network Open.*

eMethods 2. Costs and Disutility Weights

Costs and disutility weights are the same as used in recent CISNET-CRC analyses,[3,5,6] with one exception: prices of blood tests were updated to reflect the pricing of the Shield™ test.[7] For all tests. commercial costs were used for individuals younger than 65 and CMS costs were used for individuals aged 65 years and older.

Colonoscopy costs were based on an analysis of 2014 CMS data and inflated to 2021 USD using the 2021 Personal Health Care Deflator Price Index. As no data were available to inform commercial cost estimates for colonoscopy complications and CRC treatment, CMS costs were multiplied by 1.35 for individuals younger than age 65 years, based on the observed mean ratio of commercial to Medicare payment rates for colorectal tests.[8] Colonoscopy complications rates as a function of age were estimated by Hees et al.[9], using data from Warren et al.[10] Serious gastrointestinal events are perforations, gastrointestinal bleeding, or transfusions. The rate grows exponentially as a function of age, formula: 1/[exp(9.27953 - 0.06105 x Age) + 1] - 1/[exp(10.78719 - 0.06105 x Age) + 1]. Other gastrointestinal events are paralytic ileus, nausea and vomiting, dehydration, or abdominal pain. The rate depends on age, formula: 1/[exp(8.81404 - 0.05903 x Age) + 1] - 1/[exp(9.61197 - 0.05903 x Age) + 1]. Cardiovascular events are myocardial infarction or angina, arrhythmias, congestive heart failure, cardiac or respiratory arrest, syncope, hypotension, or shock. The rate depends on age, formula: 1/[exp(9.09053 - 0.07056 x Age) + 1] - 1/[exp(9.38297 - 0.07056 x Age) + 1].

FIT costs were based on 2021 Clinical Laboratory Fee Schedule data and inflated to 2021 USD using the 2021 Personal Health Care Deflator Price Index. The cost of the Shield™ test was effective May 2, 2022.[7]

Care for CRC was divided into three clinically relevant phases: initial, continuing, and terminal care. The initial care phase was defined as the first 12 months after diagnosis; the terminal care phase was defined as the final 12 months of life; the continuing care phase was defined as all months in between. In the terminal care phase, we distinguished between CRC patients dying from CRC and CRC patients dying from another cause. For patients surviving less than 24 months, the final 12 months were allocated to the terminal care phase, and the remaining months were allocated to the initial care phase.

Utility losses for life years (LYs) with initial care were derived from a study by Ness et al.[11]. For LYs with continuing care for stage I and II CRC, we assumed a utility loss of 0.05 QALYs; for LYs with continuing care for stage III and IV CRC, we assumed the corresponding utility losses for LYs with initial care. For LYs with terminal care for CRC, we assumed the utility loss for LYs with initial care for stage IV CRC. For LYs with terminal care for another cause, we assumed the corresponding utility losses for LYs with continuing care.

© 2025 Nascimento de Lima P et al. *JAMA Network Open.*

eTable. Cost and Disutility From Screening Tests, CRC Care, and Colonoscopy Complications

A. Screening tests				
Test	Commercial costs	CMS costs	Disutility when positive	Disutility when negative
Colonoscopy				
Diagnostic w/o lesion removal*	1,427.67	909.18	NA	0.000496
Surveillance w/o lesion removal	1,427.67	907.53	NA	0.000496
Any colonoscopy with lesion removal	1,889.78	1312.36	0.001401	NA
FIT	25.53	23.42	0.001330	0.000063
Blood-based test	895[d]	895	0.001330	0.000063
mtSDNA test	550	550	0.001394	0.000127
**B. Colorectal cancer care**				
2007-2013 Commercial costs per LY CRC care	Initial care	Continuing care	Terminal care Death CRC	Terminal care Death OC
Stage I CRC	55,570	78,801	114,491	170,133
Stage II CRC	5,719	6,650	10,289	48,776
Stage III CRC	112,144	126,413	132,346	166,423
Stage IV CRC	29,452	31,693	43,327	104,221
2007-2013 CMS costs per LY CRC care				
Stage I CRC	41,163	58,372	84,809	126,025
Stage II CRC	4,236	4,926	7,622	36,130
Stage III CRC	83,069	93,639	98,034	123,308
Stage IV CRC	21,816	23,476	32,094	77,200
Utility loss per LY with CRC care				
Stage I CRC	0.12	0.05	0.7	0.05
Stage II CRC	0.18	0.05	0.7	0.05
Stage III CRC	0.24	0.24	0.7	0.24
Stage IV CRC	0.7	0.7	0.7	0.7

C. Colonoscopy complications[g]			
Event	Commercial costs	CMS costs	Utility loss
Serious gastrointestinal event	11,715.00	8,677.80	0.0055
Other gastrointestinal event	8,862.00	6,564.40	0.0027
Cardiovascular event	9,541.60	7,0.67.8	0.0048

Notes: CMS = Centers for Medicare and Medicaid Services, FIT = fecal immunochemical test, CRC = colorectal cancer.

*Used for colonoscopies performed as a diagnostic follow-up after a positive non-colonoscopy test and for colonoscopies performed to diagnose symptom-detected CRC cases.

© 2025 Nascimento de Lima P et al. *JAMA Network Open.*

eReferences.

1. National Cancer Institute, DCCPS, Surveillance Research Program, Surveillance Systems Branch. Surveillance, Epidemiology, and End Results (SEER) Program (www.seer.cancer.gov) SEER*Stat Database: Incidence - SEER 22 Regs Research Data + Hurricane Katrina Impacted Louisiana Cases, Nov 2023 Sub (2000-2021). 2024.

2. Knudsen AB, Rutter CM, Peterse EFP, et al. Colorectal Cancer Screening: An Updated Modeling Study for the US Preventive Services Task Force. JAMA - Journal of the American Medical Association 2021;325:1998–2011.

3. Nascimento de Lima P, Van Den Puttelaar R, Knudsen AB, et al. Characteristics of a cost-effective blood test for colorectal cancer screening. JNCI: Journal of the National Cancer Institute 2024:djae124.

4. Nascimento de Lima P, Rutter CM, Maerzluft C, et al. Robustness Analysis of Colorectal Cancer Colonoscopy Screening Strategies. medRxiv 2023.

5. Peterse EFP, Meester RGS, Jonge L de, et al. Comparing the Cost-Effectiveness of Innovative Colorectal Cancer Screening Tests. JNCI: Journal of the National Cancer Institute 2021;113:154–161.

6. Puttelaar R van den, Lima PN de, Knudsen AB, et al. Effectiveness and Cost-Effectiveness of Colorectal Cancer Screening With a Blood Test That Meets the Centers for Medicare & Medicaid Services Coverage Decision. Gastroenterology 2024;167:368–377.

7. Guardant Health. Guardant Access. Guardant Health | Blood-Based Screening. Available at: https://bloodbasedscreening.com/guardant-access/ [Accessed July 17, 2024].

8. Ladabaum U, Mannalithara A, Brill JV, et al. Contrasting Effectiveness and Cost-Effectiveness of Colorectal Cancer Screening Under Commercial Insurance vs. Medicare. Am J Gastroenterol 2018;113:1836–1847.

9. Hees F van, Zauber AG, Klabunde CN, et al. The Appropriateness of More Intensive Colonoscopy Screening Than Recommended in Medicare Beneficiaries: A Modeling Study. JAMA Internal Medicine 2014;174:1568–1576.

10. Warren JL, Klabunde CN, Mariotto AB, et al. Adverse Events After Outpatient Colonoscopy in the Medicare Population. Ann Intern Med 2009;150:849–857.

11. Ness RM, Holmes AM, Klein R, et al. Utility valuations for outcome states of colorectal cancer. The American journal of gastroenterology 1999;94:1650–1657.

© 2025 Nascimento de Lima P et al. *JAMA Network Open.*
