# Supplementary material for: Cost-Effectiveness of Noninvasive Colorectal Cancer Screening in Community Clinics
Source: JAMA Netw Open. 2025 Jan 16;8(1):e2454938. doi: 10.1001/jamanetworkopen.2024.54938 (PMC11739995; doi:10.1001/jamanetworkopen.2024.54938)
Supplement: Supplement 2. — Data Sharing Statement [file jamanetwopen-e2454938-s002.pdf]

Data Sharing Statement

Nascimento de Lima. Cost-Effectiveness of Noninvasive Colorectal Cancer Screening in Community Clinics. *JAMA Netw Open*. Published January 16, 2025.
doi:10.1001/jamanetworkopen.2024.54938

Data
Data available: No

Additional Information
Explanation for why data not available: Code and data underlying CISNET models are not open source.
